# Advancement of the Dental Profession

**Published:** 1862-12

**Authors:** A. C. Hawes


### ADVANCEMENT OF THE DENTAL PROFESSION.

Read before the Brooklyn Dental Association, Oct. 24, 1862,

BY DR. A. C. HAWES.

*Mr. President and Gentlemen:*—The limited time allotted me, in which to obey the behests of this association, scarcely affords more than an opportunity to refer briefly to an evil that weighs heavily against the advancement of our profession, and until such steps are taken by the united efforts of its members, as will speak in tones that can not be misinterpreted, either by the fraternity or the public, we utterly fail to place our calling on the high eminence that is now being claimed for it.

*Mr. President*—I hardly need tell you, sir, that this great evil, in our very midst, is nothing more or less than the wholesale and reckless sacrifice of hundreds and of thousands of the very gems that we of the "Dental Profession" so much pride ourselves on our ability to preserve and retain for a life long service with all their original beauty and usefulness. And I assert without the remotest fear of contradiction, that this cruel sacrifice of the human teeth is not confined to the young, inexperienced, or ignorant practitioner, but that it is chargeable in a pre-eminent degree to members of the dental profession, who soar so far above the modest and less successful, but no less honest and faithful, operator that a great chasm yawns between. And, Mr. President, it is my firm conviction, that the present advanced and enlightened condition of the "healing art" entirely precludes the necessity for the extraction of ninety-nine out of every one hundred teeth that are now so ruthlessly torn from their sockets, where nature evidently designed they should remain both for ornament and use, during the natural life of man. And the dental operator of the present day, who so far forgets a plain duty, that he owes to himself, to the profession and to the public, as to sacrifice, through ignorance or cupidity, the present and the future comfort of those who have confidingly placed themselves in his hands, is deserving the severest censure, not only from this but from every Dental Association.

Mr. President, here rests a fearful responsibility, that will, I trust ere long, fall heavily on those who *still persist* in practicing this great wrong, that is now inflicting incalculable injury both to the profession and community, and I conceive it to be the duty of every member of our fraternity, who has any desire for its advancement to a higher sphere of usefulness, to put forth his best effort to check this great evil. I would therefore most respectfully suggest for the consideration of this association that some definite action be taken with especial reference to the enlightenment of the public mind; as they certainly can have no deeper interest in any branch of dental practice, than that which tends to preserve, by proper and judicious treatment,—which is now well understood by many of the profession—such teeth as are daily sacrificed by those who well "know the right yet still the wrong pursue!"

This is a subject, Mr. President, that not only requires but actually demands our serious and careful consideration, and what I assert is susceptible of the clearest proof, as the facts have been and are now daily being illustrated by many gentlemen of our profession, whose generous outpourings of the "milk of human kindness" will not allow them to retain for their own exclusive benefit and use, that which they so well know to be of inestimable value to the human family and more especially to the rising generation.

It may seem ill-timed to enumerate individual cases deserving our gratitude, when so many have shown an earnest desire to place our "speciality" on a firmer basis, but I trust that I shall be pardoned by you, Mr. President and gentlemen, if I take the liberty of mentioning the name of one of our own members, who has taken the advanced and elevated position in this matter, with all the energy and benevolence of his generous and sympathetic nature, and I assure you sir that it is with feelings of heartfelt pleasure, that I am able to say, the real friend of progress, Dr. Atkinson is with us.

Dr. Hawes is one of the rare few who do not regard expense of time or money, to be in possession of the very best knowledge in reach of the honest and honorable practitioner. He does not only talk good sense and humanity, but does the arduous work of saving the teeth condemned to the forceps, by ninety-nine hundreds of those in practice, by preparing and actually filling to the apex with solid gold, hosts of these teeth, that but for his skill, would have gone the course of all the earth, in accordance with advice of numerous, so called first class operators. These things being so as testified to by those who know him intimately, his suggestions in the above should have their full weight, and command the careful attention of every one before they perpetrate or have perpetrated upon them irreparable mischief. T.

